# Digital-Assisted Nutritional Monitoring and Body Composition Changes in Aging Adults: A 6-Month Controlled Longitudinal Study

**DOI:** 10.3390/nu18071140

**Published:** 2026-04-01

**Authors:** Rareș Gheorghe Mihuț, Timea Claudia Ghitea, Marian Morenci, Carmen Delia Cseppento Nistor, Sebastian Tirla, Diana Carina Iovanovici, Anett Karetka, Akos Tiboldi, Réka Kovács, Tünde Jurca

**Affiliations:** 1 Doctoral School of Biomedical Sciences, Faculty of Medicine and Pharmacy, University of Oradea, 410087 Oradea, Romania; mihut.raresgheorghe@student.uoradea.ro (R.G.M.); dcseppento@uoradea.ro (C.D.C.N.); tirla.sebastian@student.uoradea.ro (S.T.); balasko.anettjolan@student.uoradea.ro (A.K.); tjurca@uoradea.ro (T.J.); 2 Department of Pharmacy, Faculty of Medicine and Pharmacy, University of Oradea, 410073 Oradea, Romania; 3 Department of Psycho-Neuroscience and Recovery, Faculty of Medicine and Pharmacy, University of Oradea, 410073 Oradea, Romania; 4 Timișoara Institute of Cardiovascular Diseases, 300310 Timișoara, Romania; diana_iovanovici@yahoo.com; 5 Ludwig Boltzmann Institute for Traumatology, The Research Center in Cooperation with AUVA, 1200 Vienna, Austria; akos.tiboldi@lbg.ac.at; 6 Semmelweis University Health Services Management Training Centre, 1125 Budapest, Hungary; ko-vacs.reka@emk.semmelweis.hu

**Keywords:** digital nutrition, body composition, visceral fat, bioelectrical impedance analysis, aging, sarcopenic obesity, lifestyle intervention, remote monitoring, metabolic health

## Abstract

Background: Aging is associated with increased adiposity, sarcopenia risk, and metabolic vulnerability. Digital tools may enhance adherence to nutritional strategies, but their impact on detailed body composition remains insufficiently explored. Methods: This 6-month prospective controlled longitudinal intervention study included 60 middle-aged and older adults. All participants received a smart watch and smart scale for self-monitoring. The control group attended evaluations only at baseline and study completion, while the intervention group received monthly follow-up and remote feedback. Body composition was assessed using multi-frequency BIA. Statistical analyses included paired tests, effect sizes, correlations, and linear mixed-effects models. Results: Significant reductions were observed in BMI (*p* < 0.001), fat mass (*p* = 0.003), and visceral fat (*p* = 0.003; Cohen’s d = 0.41). The sarcopenic index improved (*p* = 0.001), while skeletal muscle mass remained stable. ECW increased significantly (*p* = 0.010). Age was inversely associated with the magnitude of improvement. Mixed-effects modeling confirmed a time-dependent reduction in visceral fat independent of age and sex. Conclusions: A 6-month digitally assisted nutritional monitoring program was associated with favorable changes in adiposity, muscle quality, and hydration status. Multi-frequency BIA provides valuable integrative markers for monitoring nutritional interventions in aging populations.

## 1. Introduction

The global rise in obesity and metabolic disorders represents a major public health challenge, particularly among middle-aged and older adults. Aging is frequently accompanied by progressive increases in fat mass, redistribution of adipose tissue toward visceral depots, and gradual declines in skeletal muscle mass and function. These changes contribute to insulin resistance, chronic low-grade inflammation, and elevated risk of cardiometabolic disease. Importantly, excess visceral adiposity has been recognized as a stronger predictor of metabolic complications than overall body weight alone [1,2,3].

Concurrently, the aging process is associated with sarcopenia and, increasingly, with sarcopenic obesity—the coexistence of excess fat mass and reduced muscle quantity or quality. This phenotype is linked to impaired physical function, frailty, and higher morbidity and mortality. Nutritional factors, including insufficient protein intake and poor dietary quality, play a central role in the development and progression of these unfavorable body composition profiles. Therefore, strategies that simultaneously address adiposity and muscle preservation are essential for healthy aging [4,5,6].

Despite increasing interest in digital lifestyle interventions, controlled longitudinal evidence combining remote nutritional monitoring with multifrequency bioelectrical impedance analysis in aging populations remains limited.

Traditional assessment of obesity has relied heavily on body mass index (BMI), which does not distinguish between fat and lean compartments or capture regional fat distribution. In this context, bioelectrical impedance analysis (BIA) has emerged as a practical and non-invasive method for evaluating body composition in both clinical and research settings. Multi-frequency BIA devices can provide detailed information on fat mass, visceral fat, skeletal muscle mass, hydration status, and phase angle, the latter being considered an indicator of cellular integrity and nutritional status. Such parameters offer a more comprehensive picture of metabolic health than BMI alone [7,8,9,10].

The adoption of wearable and non-invasive monitoring technologies has increased substantially in recent years. Surveys conducted in European and North American populations suggest that between 40% and 65% of middle-aged adults regularly use digital devices for health-related self-monitoring. Such tools have been associated with improved adherence to lifestyle interventions, although variability in long-term engagement remains a recognized limitation. In aging populations, usability and perceived clinical relevance appear to be key determinants of sustained use.

Lifestyle and nutritional interventions are the cornerstone of obesity and metabolic risk management. However, long-term adherence remains a major challenge. In recent years, digital health technologies—including smart scales, wearable devices, and remote monitoring platforms—have gained attention as tools to support behavior change. Self-monitoring of body weight and physical activity can enhance awareness, motivation, and accountability, potentially improving adherence to dietary recommendations. While several studies have examined digital tools in weight management, fewer have evaluated their impact on detailed body composition compartments, particularly in aging populations [11,12,13,14,15].

Moreover, evidence remains limited regarding how digitally supported nutritional guidance influences visceral adiposity, muscle-related parameters, and hydration status when assessed using multi-frequency BIA. Understanding these compartment-specific responses is important, as reductions in visceral fat and preservation of muscle mass may have greater clinical relevance than changes in body weight alone [16,17].

Therefore, the aim of the present study was to evaluate the effects of a 6-month digitally assisted nutritional monitoring program on body composition in middle-aged and older adults using multi-frequency BIA. We hypothesized that the intervention would be associated with reductions in adiposity—especially visceral fat—while preserving skeletal muscle mass and improving selected markers of muscle quality and hydration status.

## 2. Materials and Methods

### 2.1. Study Design and Participants

This 6-month prospective longitudinal study evaluated the effects of a digitally assisted nutritional and lifestyle monitoring program on body composition in middle-aged and older adults, from June 2024 to December 2025.

A total of 60 adults were enrolled. Eligible participants were ≥40 years of age and able to use digital monitoring devices. Exclusion criteria included acute illness, severe chronic disease with unstable clinical status, implanted electronic medical devices incompatible with bioelectrical impedance analysis (BIA), and conditions affecting fluid balance (e.g., advanced renal or hepatic disease). Participants were randomly allocated to the intervention or control group using a simple randomization procedure generated with an online random number generator (Random.org, Dublin, Ireland). Randomization was performed at enrollment by a study investigator. Group assignment was recorded prior to the start of the intervention [18].

Participants were allocated into two groups according to follow-up intensity:

- Control group—attended in-person evaluations only at baseline and at the final 6-month assessment.
- Intervention group—attended monthly follow-up visits and received continuous remote monitoring and feedback.

All participants provided written informed consent prior to inclusion.

### 2.2. Nutritional and Digital Monitoring Intervention

All participants received a smart watch and a smart scale enabling self-monitoring of body weight and physical activity throughout the study period.

The nutritional component consisted of structured dietary counseling focused on general healthy eating principles consistent with current nutritional guidelines. Participants were encouraged to:

- reduce intake of ultra-processed foods
- increase consumption of whole foods (vegetables, fruits, whole grains)
- ensure adequate protein intake to support muscle preservation
- maintain balanced macronutrient distribution
- apply moderate caloric control tailored to individual needs

The intervention group received monthly follow-up sessions that reinforced dietary guidance, reviewed device-recorded data, and supported behavioral adherence. Remote feedback promoted awareness of weight trends and physical activity patterns.

The control group received the same initial nutritional advice but did not benefit from monthly reinforcement or structured remote feedback.

No strict caloric prescription or standardized diet plan was imposed, reflecting a real-world lifestyle intervention approach.

#### Structured Nutritional Intervention Protocol

Beyond general dietary counseling, a structured macronutrient-based nutritional framework was implemented, aligned with internationally accepted acceptable macronutrient distribution ranges (AMDR) and adapted for middle-aged and older adults with pre-existing metabolic disturbances.

- The targeted macronutrient distribution was:
- Carbohydrates: 45–55% of total energy intake
- Protein: 15–30% of total energy intake
- Lipids: 15–25% of total energy intake

Carbohydrate intake prioritized low-glycemic, minimally processed sources, while added sugars and refined carbohydrates were substantially reduced. This approach aimed to improve glycemic variability and attenuate visceral adiposity progression.

Protein intake was optimized to counteract age-related anabolic resistance and support lean mass preservation. High-biological-value protein sources were encouraged, with distribution across the three principal meals to promote muscle protein synthesis efficiency.

Dietary fat intake emphasized mono- and polyunsaturated fatty acids, with restriction of saturated fats and elimination of trans-fat sources, in accordance with cardiometabolic risk reduction strategies.

Dietary fiber intake was progressively increased to an upper-optimal range of 34–42 g/day through enhanced consumption of vegetables, fruits, whole grains, and legumes. This adjustment aimed to improve satiety signaling, gut microbiota modulation, glycemic control, and systemic metabolic parameters.

Meal timing was standardized in participants presenting irregular eating patterns. A structured regimen of three principal meals per day was recommended, with snacks permitted only occasionally. This temporal restructuring targeted improved metabolic rhythm alignment and reduction of continuous energy intake behavior.

Hydration status was addressed through individualized recommendations for adequate daily water intake, adjusted to body weight and clinical status. Alcohol consumption was strongly discouraged, with a recommended target of complete abstinence (“0”), given its association with visceral adiposity and metabolic dysfunction.

No rigid caloric prescription was imposed. Instead, moderate energy control was encouraged according to individual body composition objectives and metabolic profile, preserving the pragmatic, real-world nature of the intervention.

### 2.3. Body Composition Assessment

Body composition was assessed using a multi-frequency segmental bioelectrical impedance analyzer (Tanita MC-780MA, Tanita Corporation, Tokyo, Japan), validated for clinical and research use.

The device applies low-level alternating electrical currents at multiple frequencies to estimate body composition based on tissue-specific conductivity. Using proprietary algorithms incorporating sex, age, height, and weight, the analyzer provides estimates of:

- body mass index (BMI)
- fat mass percentage
- visceral fat level
- skeletal muscle mass
- bone mass
- sarcopenic index
- extracellular water (ECW)
- ECW/total body water (ECW/TBW) ratio
- phase angle

Segmental analysis was performed for trunk and limbs.

Measurements were conducted at baseline and after 6 months under standardized conditions. Participants were measured barefoot, wearing light clothing, and instructed to:

- avoid vigorous physical activity prior to testing
- maintain usual hydration
- remove metal accessories

Each assessment lasted approximately 20 s.

### 2.4. Outcomes

The primary endpoint was change in visceral fat level between baseline and 6 months.

Secondary outcomes included changes in BMI, fat mass percentage, skeletal muscle mass, sarcopenic index (lower values indicate lower sarcopenic risk), hydration parameters (ECW and ECW/TBW), and phase angle.

### 2.5. Statistical Analysis

Statistical analyses were performed using IBM SPSS Statistics (Version 30, IBM Corp., Armonk, NY, USA).

Continuous variables are presented as mean ± standard deviation. Within-subject changes between baseline and final assessment were analyzed using paired-sample *t*-tests. Effect sizes were calculated using Cohen’s d with Hedges’ correction where appropriate.

Spearman’s rank correlation coefficient evaluated associations between age and changes in body composition parameters.

To account for repeated measures and potential confounding, a linear mixed-effects model (LMM) was applied for the primary endpoint. Time (baseline vs. final) was included as a fixed effect, with age and sex as covariates and a random intercept for each participant. Model parameters were estimated using restricted maximum likelihood (REML).

To better compare post-intervention outcomes between groups while accounting for baseline imbalance, analysis of covariance (ANCOVA) was performed for each body composition parameter. Final values were entered as dependent variables, study group as the fixed factor, and the corresponding baseline value as covariate. Age and sex were additionally included as covariates in the adjusted models.

Statistical significance was set at *p* < 0.05.

### 2.6. Ethical Considerations

The study was conducted in accordance with the Declaration of Helsinki. The protocol was approved by the institutional ethics committee of University of Oradea (Approval No. CEFMF/2 of 27 June 2024).

All participants provided written informed consent prior to participation. Data were anonymized prior to analysis.

## 3. Results

### 3.1. Baseline Characteristics

The study included 60 participants, predominantly middle-aged to older adults, with a mean age of 57.7 ± 8.5 years. The sex distribution was balanced. At baseline, the cohort exhibited a high prevalence of obesity, with a mean BMI of 33.0 ± 7.0 kg/m^2^, placing the average participant within the obese category.

Body composition analysis revealed elevated adiposity, with a mean fat mass percentage of 32.9 ± 9.3% and a mean visceral fat level of 13.3 ± 6.1 units. Skeletal muscle mass averaged 33.3 ± 7.4 kg, but with substantial variability, indicating heterogeneous muscle reserves among participants. Bone mass showed relatively low variability (3.14 ± 0.54 kg).

The sarcopenic index (8.92 ± 1.66) and phase angle (6.98 ± 2.75) suggested generally preserved muscle quality, although some individuals were already within risk ranges for sarcopenia. Body water parameters indicated elevated extracellular water (ECW 46.9 ± 6.0%) and ECW/TBW ratios (44.3 ± 3.0%), suggesting fluid imbalance and potential low-grade inflammatory or metabolic stress.

Overall, baseline data characterized a population with combined obesity-related and sarcopenia-related metabolic risk.

### 3.2. Baseline Comparison Between Control and Intervention Groups

At baseline, the two study groups were not fully comparable across all body composition parameters. Significant differences were observed for BMI, fat mass percentage, sarcopenic index, and extracellular water, while age, visceral fat, skeletal muscle mass, bone mass, ECW/TBW ratio, and phase angle did not differ significantly (Table 1).

### 3.3. Comparative Change Analysis Between the Control and Intervention Groups

Comparative analysis of change scores showed that the intervention group experienced significantly greater improvements in several adiposity-related and body composition parameters than the control group. BMI decreased by 1.51 ± 1.86 kg/m^2^ in the intervention group, whereas no change was recorded in the control group (*p* < 0.001). Fat mass decreased by 2.34 ± 3.85 percentage points in the intervention group versus 0.00 ± 0.00 in the control group (*p* = 0.002). Similarly, visceral fat was reduced by 1.33 ± 2.14 units in the intervention group, while no change was observed in controls (*p* = 0.002).

With regard to musculoskeletal parameters, skeletal muscle mass did not differ significantly between groups in terms of change over time (−0.30 ± 1.84 kg vs. 0.00 ± 0.00 kg; *p* = 0.384). However, small but statistically significant between-group differences were observed for bone mass (−0.04 ± 0.09 kg vs. 0.00 ± 0.00 kg; *p* = 0.025) and sarcopenic index (−0.29 ± 0.42 vs. 0.00 ± 0.00; *p* < 0.001). Because lower sarcopenic index values were interpreted in this dataset as favorable, this finding suggests improved muscle-related status in the intervention arm.

Hydration-related changes were more heterogeneous. ECW increased by 1.12 ± 2.18% in the intervention group compared with no change in controls, yielding a significant between-group difference (*p* = 0.008). In contrast, the change in ECW/TBW ratio did not significantly differ between groups (−0.64 ± 2.10% vs. 0.00 ± 0.00%; *p* = 0.106). Phase angle increased by 0.98 ± 3.81° in the intervention group, but this difference did not reach statistical significance compared with the control group (*p* = 0.171).

Overall, the intervention group showed a pattern of significantly greater reduction in adiposity-related parameters, particularly BMI, fat mass, and visceral fat, while structural and cellular markers showed either modest or non-significant differences relative to the control group (Table 2).

### 3.4. Within-Group Changes over the 6-Month Intervention

No relevant changes were observed in the control group between baseline and follow-up assessments. No statistically significant within-group changes were observed in the control group.

In contrast, the intervention group demonstrated consistent improvements in several adiposity-related parameters. BMI decreased from 30.21 ± 5.72 to 28.70 ± 4.71 kg/m^2^, fat mass from 30.01 ± 9.13% to 27.66 ± 8.69%, and visceral fat from 12.20 ± 6.78 to 10.87 ± 5.27 units.

Skeletal muscle mass remained relatively stable, while the sarcopenic index showed a modest reduction, suggesting potential relative changes in muscle-fat distribution rather than confirmed functional improvement. Hydration-related parameters exhibited small variations, and phase angle showed an increasing trend. Detailed values are presented in Table 3.

### 3.5. Overall Changes After Nutritional Intervention

In the overall sample, significant reductions were observed in BMI, fat mass percentage, and visceral fat after 6 months (all *p* ≤ 0.003). Skeletal muscle mass remained stable, while the sarcopenic index improved significantly.

Extracellular water distribution showed a modest but statistically significant change, whereas ECW/TBW ratio and phase angle did not reach statistical significance.

These findings indicate that the intervention primarily influenced adiposity-related compartments while maintaining structural body composition parameters (Table 4).

### 3.6. Categorical Analysis

Categorical classification analysis revealed favorable shifts in body composition categories between baseline and final assessments.

BMI categories showed a redistribution from obesity classes toward the overweight category. The proportion of overweight individuals increased from 25.0% to 35.0%, while first-degree obesity decreased from 35.0% to 26.7%.

Fat mass classification improved, with a higher proportion of participants categorized as having “good” fat mass and a reduction in the “high” category. Similarly, visceral fat distribution shifted favorably, with an increase in the “good” category (61.7% to 68.3%) and a marked reduction in the “high” category (21.7% to 11.7%).

Skeletal muscle mass categories remained largely stable, with a slight increase in participants within the normal range. Bone mass classification showed modest improvement, with fewer participants in the low bone mass category.

The sarcopenic index distribution shifted toward normal values, with the proportion of participants meeting reference ranges increasing from 16.7% to 30.0%.

Hydration-related categories also improved, with a greater proportion of participants within normal ECW and ECW/TBW ranges.

Overall, categorical analysis supports meaningful improvements in adiposity, muscle quality, and hydration status following the intervention (Table 5).

### 3.7. Age-Related Effects

Age was significantly associated with the magnitude of change in several body composition parameters.

Spearman correlation analysis demonstrated significant negative correlations between age and changes in BMI (ρ = −0.275, *p* = 0.033), fat mass percentage (ρ = −0.310, *p* = 0.016), and visceral fat (ρ = −0.332, *p* = 0.010). These findings indicate that older participants tended to experience smaller reductions in adiposity.

Similarly, age was negatively correlated with changes in skeletal muscle mass, bone mass, and sarcopenic index, suggesting reduced musculoskeletal adaptability with advancing age.

In contrast, changes in ECW, ECW/TBW ratio, and phase angle were not significantly associated with age.

**Figure 1:**
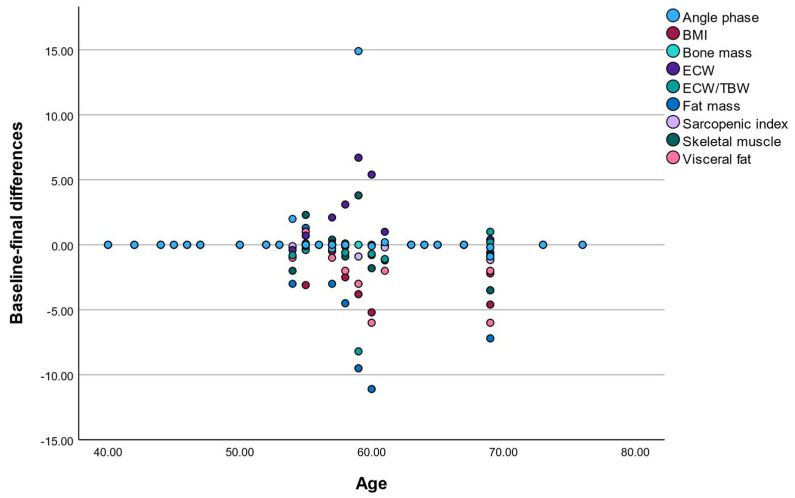
Associations between age and changes in selected body composition parameters. Scatterplots illustrate the relationships between age and changes (Δfinal–baseline). Solid lines represent linear regression trends. Older age was associated with smaller improvements in adiposity-related parameters.

These results suggest that aging may attenuate the responsiveness of adiposity and musculoskeletal parameters to nutritional and lifestyle interventions, whereas hydration and cellular health markers may be less age-dependent (Table 6 and Figure 1).

### 3.8. Primary Endpoint Analysis: Visceral Fat

Visceral fat level was prespecified as the primary endpoint. A significant within-subject reduction was observed over the 6-month period.

Mean visceral fat decreased from 13.28 ± 6.13 at baseline to 12.63 ± 5.53 at follow-up. The mean change of −0.67 units (95% CI −1.09 to −0.24) was statistically significant (*p* = 0.003) and corresponded to a moderate effect size (Cohen’s d = 0.41).

To account for repeated measures and potential confounders, a linear mixed-effects model adjusted for age and sex was applied. The model confirmed a significant time effect (β = −0.67, *p* = 0.003), indicating lower visceral fat at the final assessment independent of age and sex.

Age was positively associated with visceral fat levels in the adjusted model, while sex was not a significant predictor.

The distributional shift toward lower visceral fat values at follow-up suggests a cohort-wide effect rather than changes driven by outliers. Boxplot visualization supported a downward shift in median and upper-range visceral fat values.

**Figure 2:**
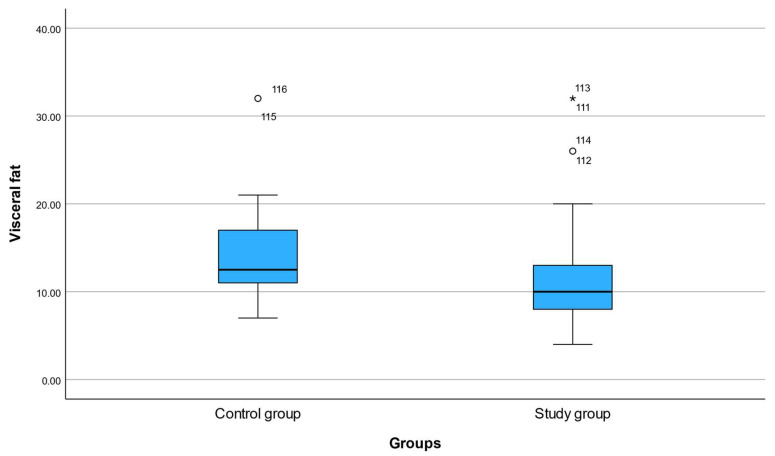
Boxplot of visceral fat levels at baseline and after the 6-month intervention. Boxes represent the interquartile range with the median indicated by the horizontal line. A significant reduction in visceral fat was observed at follow-up (*p* = 0.003). Circles denote mild outliers, while the black star (*) indicates an extreme outlier in the study group.

These findings confirm that the digitally assisted nutritional monitoring program was associated with a meaningful reduction in central adiposity (Table 7 and Figure 2).

Because baseline imbalance was observed, adjusted ANCOVA models were used to confirm between-group differences. Given these baseline imbalances, adjusted analyses were subsequently performed to better evaluate the independent effect of the digitally assisted nutritional monitoring program. Analysis of covariance (ANCOVA) models including baseline values, age, and sex as covariates were therefore applied in the between-group comparisons. ANCOVA confirmed that the intervention group had significantly lower final visceral fat values than the control group after adjustment for baseline visceral fat, age, and sex (adjusted β = −1.73, 95% CI −2.39 to −1.07, *p* < 0.001).

ANCOVA models adjusted for baseline value, age, and sex showed that, compared with the control group, the intervention group had:

- lower BMI (adjusted β = −1.94, 95% CI −2.63 to −1.25, *p* < 0.001)
- lower fat mass (adjusted β = −2.95, 95% CI −4.33 to −1.57, *p* < 0.001)
- lower visceral fat (adjusted β = −1.73, 95% CI −2.39 to −1.07, *p* < 0.001)
- no significant difference in skeletal muscle mass (adjusted β = −0.47, *p* = 0.201)
- slightly lower bone mass (adjusted β = −0.05, *p* = 0.003)
- lower sarcopenic index (adjusted β = −0.40, *p* < 0.001)
- higher extracellular water (adjusted β = +1.34, *p* = 0.003)
- no significant difference in ECW/TBW ratio (adjusted β = −0.76, *p* = 0.057)
- no significant difference in phase angle (adjusted β = +1.15, *p* = 0.109)

### 3.9. Exploratory ROC Analysis

An ROC analysis was conducted to evaluate whether baseline body composition parameters predicted a clinically meaningful reduction in visceral fat (≥1 unit). The discriminative ability of individual parameters was modest.

Baseline BMI (AUC = 0.53), fat mass percentage (AUC = 0.50), ECW/TBW ratio (AUC = 0.52), and phase angle (AUC = 0.48) showed limited predictive performance. The sarcopenic index demonstrated slightly higher discrimination (AUC = 0.58), although still within a low predictive range.

**Figure 3:**
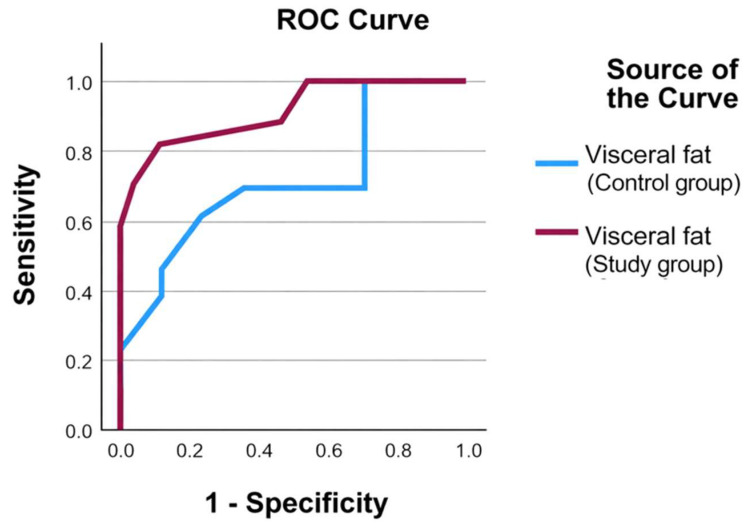
Receiver operating characteristic (ROC) curves for baseline visceral fat as a predictor of achieving a ≥1-unit reduction in visceral fat at 6 months (responder status). Sensitivity is plotted against 1 − specificity.

Overall, this exploratory analysis suggests that baseline body composition alone had limited value in identifying participants most likely to achieve visceral fat reduction. The presence of tied values may slightly influence AUC estimation (Table 8 and Figure 3).

## 4. Discussion

The present study evaluated the effects of a 6-month digitally assisted nutritional monitoring program on body composition in middle-aged and older adults using multi-frequency bioelectrical impedance analysis. The findings indicate that the intervention was associated with meaningful improvements in adiposity-related parameters, modest improvements in muscle quality indices, and partial normalization of hydration markers. Together, these results support the role of digitally supported nutritional strategies in promoting metabolic health in aging populations.

### 4.1. Adiposity Reduction and Metabolic Implications

The most consistent changes were observed in adiposity-related parameters, including BMI, fat mass percentage, and visceral fat. The significant reduction in visceral adiposity is particularly relevant, as visceral fat is strongly linked to insulin resistance, systemic inflammation, and cardiometabolic risk. Even modest decreases in visceral fat have been associated with improvements in metabolic regulation and cardiovascular risk profiles [19,20,21,22].

The 6-month duration of the intervention represents a pragmatic compromise between feasibility and expected physiological adaptation. While meaningful reductions in adiposity were observed, longer interventions may be necessary to induce consistent changes in skeletal muscle mass or cellular health markers such as phase angle.

Our findings align with previous research showing that lifestyle and dietary interventions preferentially reduce fat mass before inducing major changes in lean compartments. This pattern likely reflects the greater metabolic flexibility of adipose tissue compared with skeletal muscle in response to caloric moderation and improved dietary quality. Importantly, the moderate effect size observed for visceral fat suggests that the intervention achieved clinically meaningful changes rather than merely statistical differences. Similar reductions in visceral adiposity following moderate lifestyle interventions have been reported in recent controlled trials, supporting the clinical relevance of modest but sustained fat loss.

### 4.2. Role of Digital Monitoring and Behavioral Awareness

A distinctive feature of this study is the integration of smart devices for continuous self-monitoring. Digital tools likely contributed to improved adherence by increasing awareness of weight trends and physical activity. Behavioral research has consistently shown that self-monitoring is a powerful driver of lifestyle change, supporting self-regulation and accountability [14,23,24,25].

Monthly follow-up in the intervention group may have reinforced dietary compliance and sustained engagement. In this context, the observed improvements in adiposity may reflect not only nutritional changes but also enhanced behavioral consistency. These findings support the growing concept that digital health technologies can complement traditional nutritional counseling, particularly in long-term interventions. Previous studies have also shown that digital self-monitoring enhances adherence but may require behavioral reinforcement to maintain long-term engagement.

### 4.3. Muscle Mass, Muscle Quality, and Sarcopenic Risk

Absolute skeletal muscle mass remained largely stable, which is a favorable outcome in an aging population where muscle decline is expected. Preservation of muscle mass during fat loss is a critical goal in obesity management, especially among older adults [25,26].

Notably, the sarcopenic index improved significantly, suggesting potential enhancement in muscle quality or relative lean mass preservation. This supports the idea that adequate protein intake and balanced nutrition may help mitigate sarcopenia risk during weight management interventions. Rather than inducing large anabolic gains, realistic nutritional strategies in older adults may primarily slow muscle loss and improve muscle function.

### 4.4. Hydration Status and Cellular Health

Changes in body water distribution provide additional insight into metabolic adaptation. A small but significant change in ECW was observed suggests a shift toward improved fluid balance and possibly reduced inflammatory burden. Elevated ECW is frequently associated with metabolic dysfunction and low-grade inflammation; therefore, its partial normalization may reflect systemic benefits of dietary improvement [27,28,29]. The increase in ECW may reflect physiological fluid redistribution rather than a purely beneficial adaptation.

Phase angle showed a non-significant upward trend, with substantial inter-individual variability. Because phase angle reflects cellular membrane integrity and body cell mass, longer interventions or more intensive nutritional strategies may be required to detect consistent changes in this parameter.

### 4.5. Influence of Age on Responsiveness

Age emerged as a moderator of intervention response. Older participants showed smaller improvements in adiposity and musculoskeletal parameters, consistent with reduced metabolic plasticity and anabolic resistance. Aging is associated with hormonal changes, chronic inflammation, and decreased adaptive capacity, which may limit the magnitude of body composition changes [30,31,32,33,34].

Interestingly, hydration and phase angle changes were less strongly related to age, suggesting that certain physiological domains may remain modifiable even in older individuals. This highlights the importance of early and sustained interventions before advanced age-related decline occurs. Age-related anabolic resistance and reduced metabolic plasticity have been consistently described as factors limiting body composition responsiveness in older adults.

### 4.6. Clinical and Public Health Relevance

From a practical perspective, the findings underscore that meaningful improvements in body composition can be achieved through moderate, digitally supported nutritional strategies without extreme dietary restriction. The combination of nutritional guidance and digital self-monitoring represents a scalable and accessible approach, particularly relevant in aging societies where chronic disease burden is rising [35,36,37].

Multi-frequency BIA proved useful for capturing subtle changes across multiple compartments, offering a more nuanced assessment than body weight alone. Such tools may help clinicians personalize interventions and monitor progress beyond simple anthropometric measures.

Although multi-frequency BIA provides a practical and non-invasive method for monitoring body composition in real-world settings, it remains an indirect technique influenced by hydration status, recent physical activity, and device-specific algorithms. Therefore, the results should be interpreted cautiously, and future studies may benefit from complementary imaging or biochemical assessments.

### 4.7. Limitations

Several limitations should be acknowledged. The sample size was moderate, and the study was not designed as a fully randomized controlled trial, which may limit causal inference. Dietary intake was guided but not rigorously quantified, preventing precise evaluation of nutrient-level effects. Physical activity was monitored but not standardized. Additionally, biochemical markers of metabolic health were not included, which restricts mechanistic interpretation.

It should also be noted that no relevant changes were detected in the control group across the study period. This pattern may reflect limited adherence to lifestyle recommendations in the absence of structured monitoring, but it may also be influenced by methodological factors such as measurement timing, inter-individual variability, and the observational nature of the control condition. Consequently, caution is warranted when interpreting the magnitude of the intervention effect.

The absence of measurable change in the control group may partly reflect limited behavioral reinforcement, but may also be influenced by methodological factors such as measurement timing, biological variability, and the pragmatic observational nature of the control condition. The absence of complementary assessment techniques represents an additional limitation. Future studies should consider the use of reference imaging methods such as DEXA or MRI to improve the accuracy of visceral fat and muscle mass estimation. Objective physical activity monitoring through accelerometry and the inclusion of metabolomic and biochemical markers would also help clarify the mechanisms underlying observed body composition changes.

Future studies should incorporate advanced imaging methods such as DEXA or MRI to improve the precision of body composition assessment. Objective monitoring of physical activity using accelerometry and the inclusion of metabolomic and biochemical markers would also provide deeper insight into the physiological mechanisms underlying the observed changes.

Overall, our results suggest that digitally assisted nutritional monitoring represents a promising strategy for improving adiposity and metabolic resilience in middle-aged and older adults.

## 5. Conclusions

This study demonstrates that a 6-month digitally assisted nutritional monitoring program was associated with favorable changes in body composition in middle-aged and older adults. The most consistent improvements were observed in adiposity-related parameters, particularly visceral fat, while skeletal muscle mass was largely preserved and muscle quality indices showed modest enhancement. These findings suggest that moderate nutritional guidance supported by digital self-monitoring can promote healthier body composition without compromising lean tissue in aging populations.

The integration of smart devices and regular follow-up may enhance behavioral adherence and support sustainable lifestyle changes. Multi-frequency bioelectrical impedance analysis proved to be a practical tool for monitoring compartment-specific responses beyond body weight alone.

Overall, digitally supported nutritional strategies represent a promising and scalable approach for reducing central adiposity and improving metabolic resilience in midlife and older age. Future research should incorporate randomized designs, detailed dietary assessment, and metabolic biomarkers to further clarify long-term clinical benefits.

## Figures and Tables

**Table 1:** Baseline characteristics according to study group.

Parameter	Control Group (N = 30)	Intervention Group (n = 30)	*p*-Value
Age (years)	57.47 ± 9.00	57.90 ± 8.13	0.845
Female, n (%)	17 (56.7)	13 (43.3)	0.302
BMI (kg/m^2^)	35.99 ± 7.02	30.21 ± 5.72	**0.001**
Fat mass (%)	35.85 ± 8.53	30.01 ± 9.13	**0.013**
Visceral fat (level)	14.40 ± 5.30	12.20 ± 6.78	0.167
Skeletal muscle mass (kg)	34.45 ± 7.54	32.14 ± 7.23	0.230
Bone mass (kg)	3.25 ± 0.49	3.04 ± 0.57	0.124
Sarcopenic index	9.45 ± 1.59	8.42 ± 1.55	**0.014**
ECW (%)	44.95 ± 5.85	48.89 ± 5.61	**0.010**
ECW/TBW (%)	44.84 ± 3.27	43.72 ± 2.66	0.150
Phase angle (°)	6.58 ± 1.56	7.29 ± 3.55	0.322

Values are presented as mean ± standard deviation unless otherwise specified. Between-group comparisons were performed using independent samples *t*-tests or χ^2^ tests where appropriate. Significant differences are shown in bold.

**Table 2:** Comparative change analysis of body composition parameters between the control and intervention groups over 6 months.

Parameter	Control Group Δ (Final–Baseline)	Intervention Group Δ (Final–Baseline)	*p*-Value (Between Groups)
BMI (kg/m^2^)	0.00 ± 0.00	−1.51 ± 1.86	<0.001
Fat mass (%)	0.00 ± 0.00	−2.34 ± 3.85	0.002
Visceral fat (level)	0.00 ± 0.00	−1.33 ± 2.14	0.002
Skeletal muscle mass (kg)	0.00 ± 0.00	−0.30 ± 1.84	0.384
Bone mass (kg)	0.00 ± 0.00	−0.04 ± 0.09	0.025
Sarcopenic index	0.00 ± 0.00	−0.29 ± 0.42	<0.001
ECW (%)	0.00 ± 0.00	+1.12 ± 2.18	0.008
ECW/TBW (%)	0.00 ± 0.00	−0.64 ± 2.10	0.106
Phase angle (°)	0.00 ± 0.00	+0.98 ± 3.81	0.171

Values are presented as mean ± standard deviation of change scores (final minus baseline). Between-group comparisons were performed using independent-samples tests on Δ values. Negative values indicate reductions over time.

**Table 3:** Changes in anthropometric and body composition parameters after the 6-month intervention according to study group.

Parameter	Control Group Baseline	Control Group Final	Intervention Group Baseline	Intervention Group Final
BMI (kg/m^2^)	35.99 ± 7.02	35.99 ± 7.02	30.21 ± 5.72	28.70 ± 4.71
Fat mass (%)	35.85 ± 8.53	35.85 ± 8.53	30.01 ± 9.13	27.66 ± 8.69
Visceral fat (level)	14.40 ± 5.30	14.40 ± 5.30	12.20 ± 6.78	10.87 ± 5.27
Skeletal muscle mass (kg)	34.45 ± 7.54	34.45 ± 7.54	32.14 ± 7.23	31.84 ± 6.24
Bone mass (kg)	3.25 ± 0.49	3.25 ± 0.49	3.04 ± 0.57	3.00 ± 0.53
Sarcopenic index	9.45 ± 1.59	9.45 ± 1.59	8.42 ± 1.55	8.13 ± 1.36
ECW (%)	44.95 ± 5.85	44.95 ± 5.85	48.89 ± 5.61	50.01 ± 5.47
ECW/TBW (%)	44.84 ± 3.27	44.84 ± 3.27	43.72 ± 2.66	43.08 ± 2.75
Phase angle (°)	6.58 ± 1.56	6.58 ± 1.56	7.29 ± 3.55	8.27 ± 4.90

**Table 4:** Changes in anthropometric and body composition parameters after the 6-month digital-assisted nutritional intervention (N = 60).

Parameter	Baseline Mean ± SD	Final Mean ± SD	Mean Difference	*p*-Value	Cohen’s d
Adiposity parameters					
BMI (kg/m^2^)	33.00 ± 7.05	32.34 ± 6.97	−0.76	<0.001	0.50
Fat mass (%)	32.88 ± 9.27	31.76 ± 9.48	−1.17	0.003	0.40
Visceral fat (level)	13.28 ± 6.13	12.63 ± 5.53	−0.67	0.003	0.41
Musculoskeletal parameters					
Skeletal muscle mass (kg)	33.29 ± 7.42	33.14 ± 6.99	−0.15	0.380	0.11
Bone mass (kg)	3.14 ± 0.54	3.13 ± 0.52	−0.02	0.027	0.29
Sarcopenic index	8.92 ± 1.66	8.79 ± 1.61	−0.15	0.001	0.45
Hydration parameters					
ECW (%)	46.94 ± 6.01	47.48 ± 6.17	+0.56	0.010	−0.35
ECW/TBW (%)	44.27 ± 3.03	43.96 ± 3.13	−0.32	0.106	0.21
Cellular health					
Phase angle (°)	6.98 ± 2.75	7.43 ± 3.71	+0.49	0.169	−0.18

Values are presented as mean ± standard deviation. Mean difference calculated as final–baseline. *p*-values derived from paired-sample *t*-tests. Cohen’s d interpreted as: trivial (<0.2), small (0.2–0.49), moderate (0.5–0.79), large (≥0.8).

**Table 5:** Distribution of participants across body composition categories at baseline and after the 6-month intervention (N = 60).

Parameter	Category	Baseline n (%)	Final N (%)
BMI	Normal	5 (8.3)	5 (8.3)
	Overweight	15 (25.0)	21 (35.0)
	Obesity class I	21 (35.0)	16 (26.7)
	Obesity class II	12 (20.0)	11 (18.3)
	Obesity class III	7 (11.7)	7 (11.7)
Fat mass	Low	1 (1.7)	1 (1.7)
	Normal/Good	13 (21.7)	19 (31.7)
	Increased	19 (31.7)	18 (30.0)
	High	27 (45.0)	22 (36.7)
Visceral fat	Normal	37 (61.7)	41 (68.3)
	Increased	10 (16.7)	12 (20.0)
	High	13 (21.7)	7 (11.7)
Skeletal muscle mass	Normal	20 (33.3)	21 (35.0)
	High	40 (66.7)	39 (65.0)
Bone mass	Low	14 (23.3)	12 (20.0)
	Normal	46 (76.7)	48 (80.0)
Sarcopenic index	Normal	10 (16.7)	18 (30.0)
	Above reference	50 (83.3)	42 (70.0)
ECW	Low	36 (60.0)	32 (53.3)
	Normal	24 (40.0)	28 (46.7)
ECW/TBW	Normal	5 (8.3)	10 (16.7)
	High	55 (91.7)	50 (83.3)

Categories were defined according to manufacturer reference ranges for the Tanita MC-780MA analyzer. Percentages are calculated within the total sample (N = 60).

**Table 6:** Spearman correlations between age and changes in body composition parameters (Δfinal–baseline) (N = 60).

Parameter (ΔChange)	Spearman’s ρ	*p*-Value
ΔBMI	−0.275	0.033 *
ΔFat mass (%)	−0.310	0.016 *
ΔVisceral fat	−0.332	0.010 **
ΔSkeletal muscle mass	−0.256	0.048 *
ΔBone mass	−0.294	0.023 *
ΔSarcopenic index	−0.301	0.020 *
ΔECW (%)	0.104	0.431
ΔECW/TBW (%)	0.103	0.435
ΔPhase angle	−0.153	0.243

Δ change calculated as final minus baseline values. * *p* < 0.05, ** *p* < 0.01.

**Table 7:** Primary endpoint analysis: Changes in visceral fat over the 6-month intervention (N = 60).

Analysis Component	Result
Descriptive statistics	
Baseline visceral fat (mean ± SD)	13.28 ± 6.13
Final visceral fat (mean ± SD)	12.63 ± 5.53
Mean change (Δfinal–baseline)	−0.67
95% CI of change	−1.09 to −0.24
Paired-sample analysis	
t (df = 59)	3.14
*p*-value	0.003
Cohen’s d	0.41 (moderate)
Linear mixed-effects model	
Time effect (β)	−0.67
95% CI	−1.09 to −0.24
*p*-value	0.003
Age effect (β)	0.04
*p*-value (age)	0.021
Sex effect (β)	0.58
*p*-value (sex)	0.140
Model fit	
AIC	420.6
BIC	432.1

Mean change calculated as final minus baseline. Negative values indicate reduction. Linear mixed-effects model included time, age, and sex as fixed effects with a random intercept for participants. Statistical significance set at *p* < 0.05.

**Table 8:** ROC analysis for prediction of visceral fat responders.

Baseline Parameter	AUC
BMI	0.53
Fat mass (%)	0.50
ECW/TBW	0.52
Phase angle	0.48
Sarcopenic index	0.58

AUC, area under the receiver operating characteristic (ROC) curve. Responders were defined as participants with a reduction in visceral fat ≥1 unit from baseline to follow-up. ROC analyses assessed the discriminative ability of baseline body composition parameters. An AUC of 0.5 reflects no discriminative capacity, while values approaching 1.0 indicate stronger predictive performance.

## Data Availability

Data of the patients are available in the medical archive of the Echo Laboratories.
